# Acute Periprosthetic Joint Infection After Primary Total Knee Arthroplasty: Experience From a Secondary Public Hospital in Northern Mexico

**DOI:** 10.7759/cureus.112273

**Published:** 2026-07-08

**Authors:** Diego Emmanuel Almeida-Muñoz, Alexis Valenzuela-González, Ruben Cuevas-Martínez

**Affiliations:** 1 Research, Hospital General Dr Salvador Zubirán Anchondo, Chihuahua, MEX; 2 Orthopedics and Traumatology, Hospital General Dr Salvador Zubirán Anchondo, Chihuahua, MEX

**Keywords:** 2018 msis criteria, incidence and risk factors, mexico, periprosthetic joint infection (pji), primary total knee arthroplasty, surgical site infection(ssi)

## Abstract

Background

Periprosthetic joint infection (PJI) is an uncommon but serious complication of total knee arthroplasty (TKA), and local data from secondary-level public hospitals in Mexico are limited. We measured the incidence of acute PJI after primary TKA at our hospital, described the patients who developed it, and established a local baseline for prevention and surveillance.

Methods

We retrospectively reviewed the medical records of 104 consecutive knee arthroplasty patients from October 2024 to September 2025. After excluding one revision and one bilateral procedure, 102 primary unilateral TKAs performed for osteoarthritis were analyzed. Acute PJI was defined by the Musculoskeletal Infection Society (MSIS)/2018 International Consensus Meeting and Infectious Diseases Society of America (IDSA) criteria. Cohort characteristics and the incidence of acute PJI (with a 95% confidence interval (CI)) were summarized descriptively. With only two events, we did not attempt inferential risk-factor analysis; instead, we describe both cases in detail.

Results

Two patients developed acute PJI, an incidence of 2.0% (2/102; 95% CI: 0.5-6.9%). The cohort was 56.9% female, with diabetes in 24.5%, hypertension in 44.1%, smokers in 5.9%, preoperative anemia in 13.7%, and no perioperative transfusions. One case was culture-negative with a sinus tract communicating with the joint (a major criterion); the other grew *Staphylococcus aureus*. Both patients had normal preoperative hemoglobin, required infection-related readmissions (7 and 29 days), and had infections that resolved after debridement, targeted antibiotics, and implant retention, with cultures turning negative and clinical signs resolving by one month and six weeks, respectively, and no recurrence over 12 months of follow-up.

Conclusions

This study establishes our hospital's first institutional baseline for acute knee PJI after primary TKA. With only two events, the estimate is imprecise (95% CI: 0.5-6.9%) and precludes risk-factor inference, but it supports a guideline-based prevention and surveillance algorithm, a prospective registry, and future adequately powered multicenter studies.

## Introduction

Total knee arthroplasty (TKA) relieves pain and restores function in severe knee osteoarthritis, and its use continues to increase. Infection of the prosthesis is uncommon, but when it occurs, it is among the most severe complications, requiring reoperation, prolonged morbidity, and increased mortality [1,2]. Roughly one million hip and knee replacements are performed each year in the United States; periprosthetic joint infection (PJI) affects about 1-2% of them and is now the leading reason for revision surgery [3].

The microorganisms responsible settle on the implant and build a biofilm that shields them from both the immune system and antibiotics, which renders these infections difficult to eradicate. *Staphylococcus aureus* and coagulase-negative *staphylococci* such as *S. epidermidis* are the most common causative organisms [3,4]. No single test confirms the diagnosis; current practice combines the Musculoskeletal Infection Society (MSIS)/2018 International Consensus Meeting and Infectious Diseases Society of America (IDSA) criteria, and infection is classified as acute (early postoperative) when it presents within 90 days of surgery, as distinct from delayed and chronic infection [5,6].

When the implant is stable and the infection is caught early, debridement, antibiotics, and implant retention (DAIR) can preserve the prosthesis [7]. Prevention depends on identifying modifiable risk factors. Diabetes and poor glycemic control, obesity, smoking, malnutrition, preoperative anemia, and perioperative transfusion have all been associated with higher infection rates, the latter likely through transfusion-related immunomodulation [4,8-10]. The Mexican setting largely lacks data from secondary-level public hospitals, where much of the country's arthroplasty is performed. Because infection rates, perioperative practices, and patient populations differ across healthcare settings, institution-specific data are needed to guide local prevention rather than relying on figures from better-resourced centers. Our primary objective was to determine the incidence of acute PJI after primary TKA at our hospital. The secondary objectives were to describe the clinical characteristics of the patients who developed it and to establish a local baseline to guide prevention and surveillance.

## Materials and methods

This was a single-center retrospective cohort study at the Hospital General "Dr. Salvador Zubirán Anchondo," a secondary-level public hospital in Chihuahua, Mexico, including procedures performed between October 2024 and September 2025. Reporting follows the Strengthening the Reporting of Observational Studies in Epidemiology (STROBE) recommendations.

**Figure 1:**
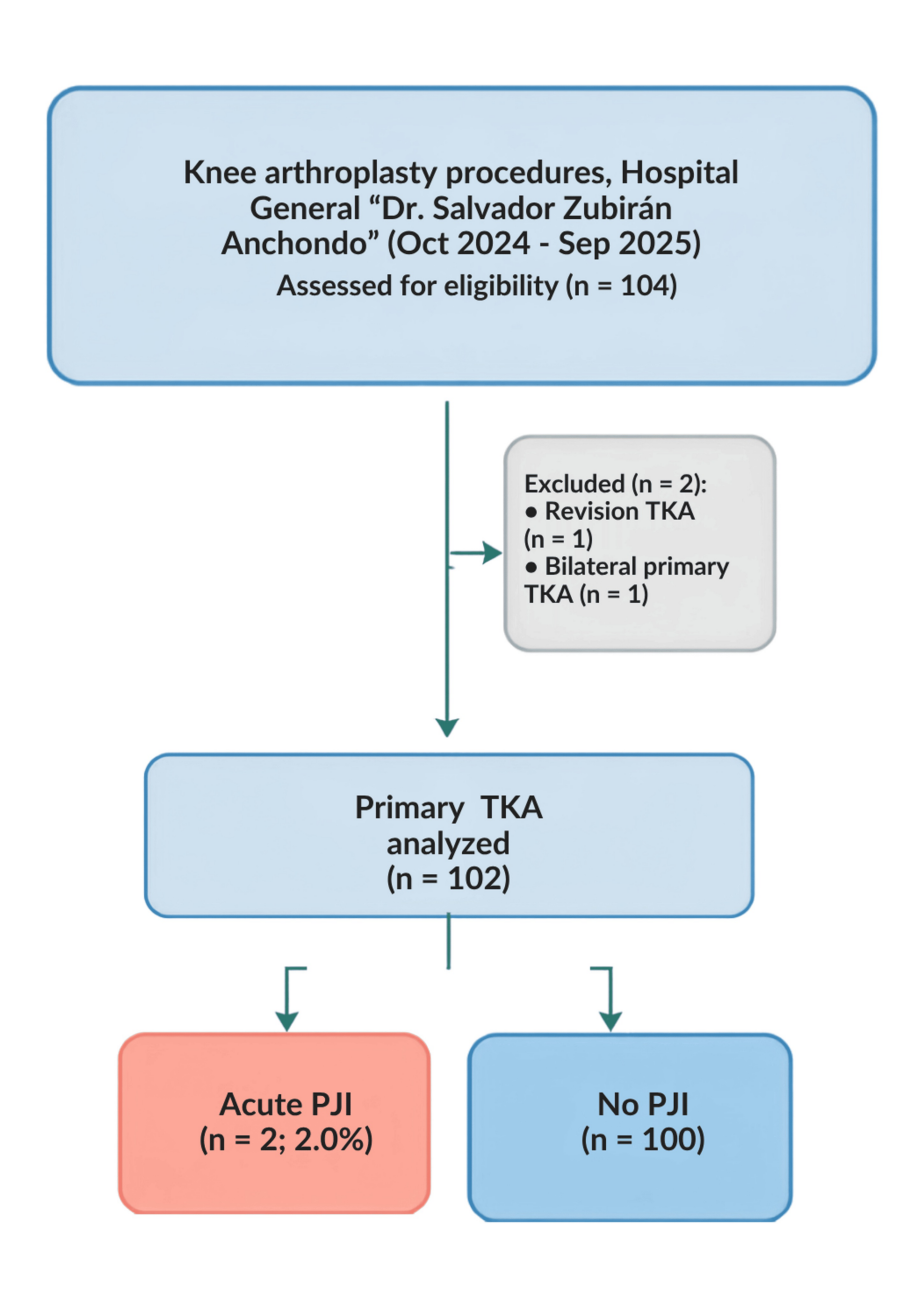
Patient selection flow diagram (Strengthening the Reporting of Observational Studies in Epidemiology (STROBE)). Flow of knee arthroplasty records through the study. Of 104 consecutive knee arthroplasty records identified between October 2024 and September 2025, one revision and one bilateral procedure were excluded, leaving 102 primary unilateral TKAs performed for osteoarthritis for analysis. TKA: total knee arthroplasty.

We included every consecutive patient admitted under trauma and orthopaedics for primary unilateral TKA performed for osteoarthritis. We excluded bilateral primary TKA, revision TKA, non-osteoarthritis indications, and in-hospital death. Patients with a clinical suspicion of infection who did not meet the MSIS/2018 International Consensus Meeting criteria - whether by a positive culture or by other major or minor criteria - were not classified as PJI; culture-negative infections that met other criteria were retained (one of our two cases), so this conservative rule applied only to unconfirmed suspicion, a point revisited in the Limitations. Of 104 knee arthroplasty records identified over the period, one revision and one bilateral procedure were excluded, leaving 102 patients for analysis (Figure 1). Acute PJI was ascertained from the institutional clinical record, including emergency visits and readmissions. Because the outcome was defined within the first 90 postoperative days, all 102 patients had complete follow-up for the acute period; per institutional protocol, patients are followed for a minimum of 12 months, an interval not yet completed by those operated toward the end of the study period. No a priori sample size calculation was performed; as a descriptive study, the sample comprised all consecutive eligible patients treated during the study period rather than a number selected to detect a predefined effect size.

The outcome was acute PJI, defined by the MSIS/2018 International Consensus Meeting major and minor criteria together with the IDSA framework, and was considered acute when it occurred within 90 days of the index arthroplasty (early postoperative period) [6]. We recorded age, sex, diabetes, hypertension, smoking, preoperative anemia, perioperative transfusion, surgical time, length of stay, antibiotic prophylaxis, wound complications, and the isolated pathogen when applicable. Antibiotic prophylaxis followed the institutional protocol: ceftriaxone 1 g intravenously one hour before surgery, followed by ciprofloxacin 500 mg orally daily for 10 days after surgery.

Categorical variables are given as counts and proportions, and continuous variables as mean and standard deviation (SD). One age value was missing and was handled by complete-case analysis (n = 101) without imputation; no other variable had missing data. Preoperative anemia was defined per WHO criteria as hemoglobin below 12 g/dL in women and 13 g/dL in men. Incidence is reported as a proportion with a 95% Wilson confidence interval (CI). Because only two infections occurred, we did not calculate odds ratios or relative risks: with so few events, these estimates would be unstable, carry CIs spanning several orders of magnitude, and could not be adjusted for confounding. We describe the two confirmed cases individually and leave formal risk-factor estimation for a larger, adequately powered study.

The study was approved by the Research Ethics Committee of the Hospital General "Dr. Salvador Zubirán Anchondo" (registration CONBIOETICA-08-CEI-001-20170517; approval letter CEI-AP-0042-2025, dated December 9, 2025; protocol registry folio 0422, Volume IV).

This retrospective review examined clinical records only and was classified as minimal-risk research; individual informed consent was not required. Data were used for scientific purposes only.

## Results

Of the 102 patients who underwent primary TKA for osteoarthritis, two developed acute PJI, for an incidence of 2.0% (2/102; 95% CI 0.5-6.9%) - an infrequent event at our center, broadly in keeping with international series, although with only two events, this estimate is imprecise. No patient in the analyzed cohort received a perioperative transfusion; antibiotic prophylaxis was administered in all cases, and no wound complications were recorded during follow-up. The index surgical admission was uniformly short (mean 2.0 days, SD 0.2; range 1-3). The cohort's characteristics appear in Table 1.

**Table 1:** Baseline characteristics of the cohort (N = 102). Preoperative anemia is defined per WHO criteria (hemoglobin <12 g/dL in women, <13 g/dL in men). SD: standard deviation.

Characteristic	Value
Categorical variables, n (%)	
Female sex	58 (56.9)
Male sex	44 (43.1)
Diabetes mellitus	25 (24.5)
Arterial hypertension	45 (44.1)
Active smoking	6 (5.9)
Preoperative anemia*	14 (13.7)
Perioperative blood transfusion	0 (0)
Antibiotic prophylaxis	102 (100)
Acute periprosthetic joint infection	2 (2.0)
Continuous variables, mean (SD)	
Age, years	64.9 (7.6)
Preoperative hemoglobin, g/dL	13.4 (0.8)
Surgical time, minutes	88.5 (13.6)
Length of index admission, days	2.0 (0.2)

Both infections were managed with DAIR; the clinical signs of infection resolved with wound healing, closure of the sinus tract, and normalization of inflammatory markers by one month and six weeks, respectively, and no recurrence over 12 months of follow-up. The two infected patients had uncomplicated two-day index admissions and were subsequently readmitted for infection management (7 and 29 days); these readmissions, not the index stay, account for their prolonged total hospitalization. The second case grew *S. aureus*; the first was culture-negative but met a major criterion through a sinus tract communicating with the joint. Table 2 details the course of each case.

**Table 2:** Clinical characteristics and course of the two acute periprosthetic joint infection (PJI) cases. Index surgical admission was two days in both cases; durations shown are infection-related readmissions.

Feature	Case 1	Case 2
Age, sex	63, female	62, male
Infection-related readmission, days*	7	29
Preoperative hemoglobin	13.7 g/dL	13.7 g/dL
Comorbidities	Hypertension	None recorded
Microbiology	Negative wound culture; normal procalcitonin	*Staphylococcus aureus*; fetid purulent drainage communicating with the prosthesis
Diagnostic basis	Sinus tract communicating with the joint (major criterion); local warmth, swelling, and pain on mobilization; and leukocytosis	Positive culture and purulent drainage meeting the 2018 ICM/MSIS threshold through minor criteria; local warmth, swelling, and pain on mobilization
Management	Surgical washout and sinus-tract excision; oral antibiotics; and implant retained	Debridement of non-viable tissue; targeted antibiotics; and implant retained
Outcome	Wound healed and sinus tract closed; inflammatory markers normalized by one month; no recurrence at 12-month follow-up	Wound healed with negative repeat cultures and normalized inflammatory markers by six weeks; no recurrence at 12-month follow-up

## Discussion

Among 102 patients who underwent primary unilateral TKA at our secondary-level hospital, two developed acute PJI, an incidence of 2.0%. This falls within the 0.7-2% range reported nationally and internationally for centers with standardized perioperative protocols [9,11]. It aligns with the 0.7% reported by Pulido et al. in a far larger cohort [9] and comes close to the 1.79% described by Franco-Cendejas et al. at a specialized center in Mexico City [12]. However, with only two infections among 102 patients, the estimate is imprecise (95% CI 0.5-6.9%) and should be interpreted with caution; it cannot be taken as evidence of adequate perioperative control, which this descriptive, single-center study was not designed to assess.

The rarity of the outcome is the study's principal analytical constraint. Two cases leave essentially no room for inference; we therefore did not report odds ratios or relative risks, which, with so few events, would be highly unstable and uninformative. The results are best interpreted as descriptive and hypothesis-generating rather than as confirmation of any association, consistent with PJI research, where even multicenter cohorts require large samples to identify independent risk factors [6,13].

Neither infected patient was diabetic, a smoker, or transfused, and both had normal preoperative hemoglobin levels; the anemia documented during their infection-related readmissions therefore developed after surgery rather than before it. Both required prolonged stays (7 and 29 days), but those admissions were largely the time needed to treat the infection itself, so they cannot be read as a clean antecedent. We mention these features only to describe the two cases, not to claim associations. In the broader literature, preoperative anemia, allogeneic transfusion, and prolonged admission have each been linked to PJI risk through immunomodulation, impaired tissue oxygenation, and greater exposure to hospital-acquired organisms [9,10,14]. In our cohort, no patient received a perioperative transfusion, and 13.7% had preoperative anemia. These baseline rates provide a reference point that larger studies could build on. Microbiology followed the expected pattern: *S. aureus* in one case and a culture-negative sinus-tract infection in the other. Both resolved after early debridement and directed antibiotics, without recurrence at 12 months, underscoring the value of timely diagnosis and guideline-based care [3,5,15].

**Figure 2:**
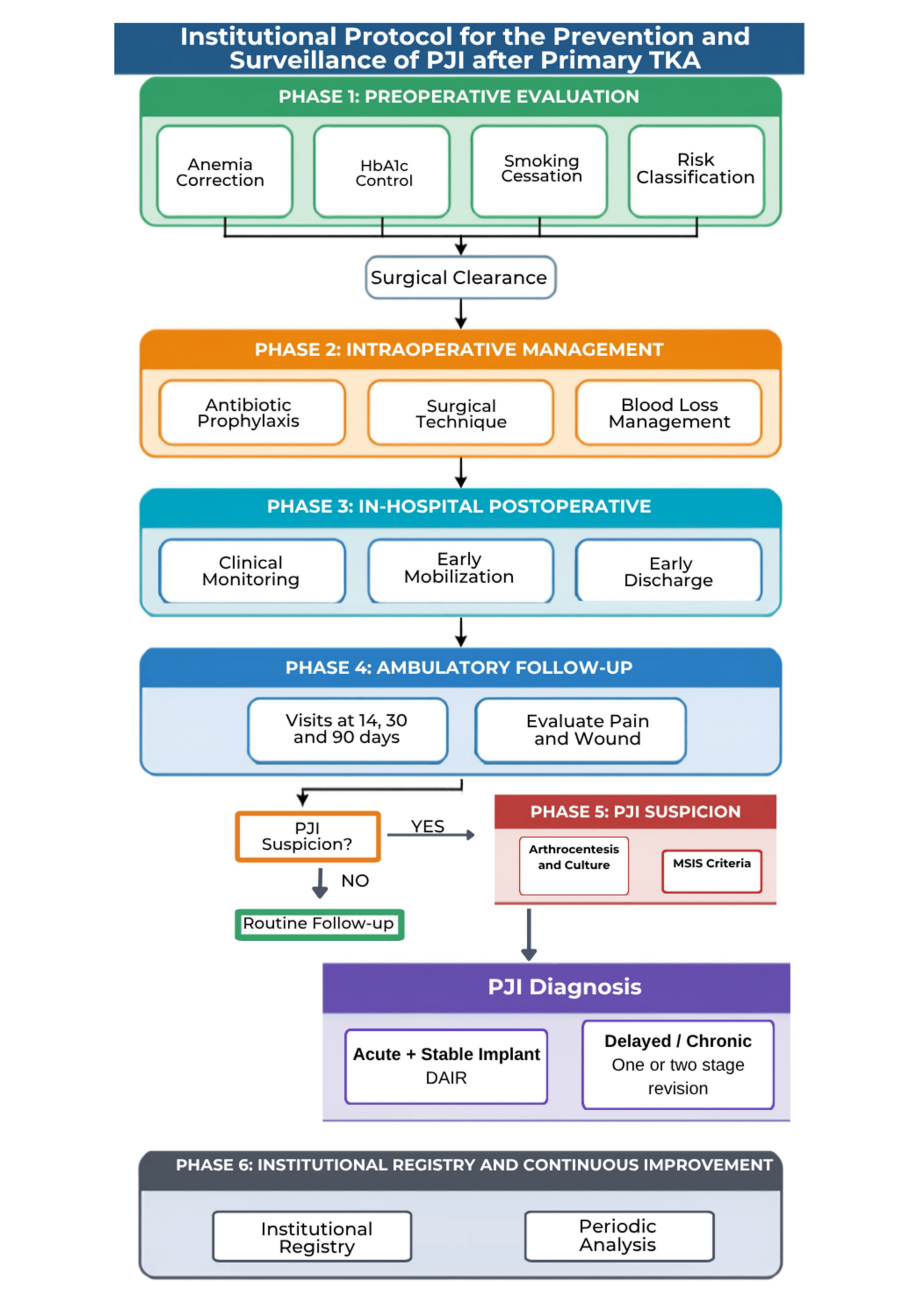
Guideline-based institutional algorithm for the prevention and surveillance of PJI after primary TKA. The pathway is grounded in International Prevention Guidelines (Allegranzi et al. 2016; Shohat & Parvizi 2017) rather than derived from the two observed cases; the institutional baseline incidence provides the rationale for its implementation and prospective audit. Phase 1 addresses preoperative optimization (anemia correction, glycemic control by HbA1c, smoking cessation, and risk classification); Phases 2-3 cover intraoperative and in-hospital measures; and Phase 4 provides structured ambulatory follow-up at 14, 30, and 90 days. When PJI is suspected (Phase 5), diagnosis follows MSIS/2018 ICM criteria: acute infection with a stable, well-fixed implant is managed by DAIR, whereas delayed or chronic PJI generally requires one- or two-stage revision. Phase 6 supports a prospective institutional registry and continuous quality improvement. TKA: total knee arthroplasty.

The principal contribution of this study is an institutional baseline rate of acute knee PJI that was not previously available. This baseline does not, by itself, identify local risk factors; it provides the rationale for a structured, guideline-based prevention and surveillance pathway. Accordingly, the algorithm we propose (Figure 2) is grounded in established international prevention recommendations [16,17] rather than derived from our two cases. It comprises preoperative optimization (correction of anemia, glycemic control assessed by glycated hemoglobin (HbA1c), and smoking cessation), guideline-concordant antibiotic prophylaxis, restrictive transfusion, early mobilization, and structured follow-up at 14, 30, and 90 days, together with a prospective arthroplasty registry to track outcomes, complications, and pathogens over time. When PJI is suspected, management follows accepted criteria: DAIR applies to acute infection with a stable, well-fixed implant, whereas delayed or chronic PJI generally requires one- or two-stage revision [7].

This study has several limitations, most of which follow from its size and its retrospective, single-center design. With only two infections among 102 patients, the incidence estimate is imprecise (95% CI: 0.5-6.9%) and should be read as a first local approximation rather than a precise rate; it does not support conclusions about the quality of perioperative care and rules out multivariable adjustment or any claim about independent risk factors. As a retrospective study, it relied on existing clinical records, with the missing data noted above. Several variables relevant to infection risk were not captured - body mass index and obesity, glycated hemoglobin, nutritional status, nasal *S. aureus* carriage, and ASA physical status classification - which limits interpretation of the prevention targets we propose; diabetes, for example, was recorded only as a binary variable. Because patients with clinical suspicion of infection who did not meet standardized diagnostic criteria were not classified as PJI, and because infections treated at outside hospitals after discharge may not have been captured, the true incidence could be underestimated. The antibiotic prophylaxis used in our public hospital setting (ceftriaxone with a 10-day postoperative oral course) reflects locally available agents and differs from current guideline recommendations, which favor a single preoperative dose of a first-generation cephalosporin discontinued within 24 hours [16]; rationalizing prophylaxis is one of the quality-improvement targets our registry is intended to support. Finally, follow-up was oriented to the acute postoperative period; although the institutional protocol targets a minimum of 12 months - not yet completed by the most recently operated patients - this window would not detect delayed or chronic PJI, which can present later.

## Conclusions

Acute PJI occurred in 2.0% of patients after primary TKA at our hospital, and both infections resolved after DAIR, without recurrence over 12 months of follow-up. Although limited by the small number of events, this study establishes the first institutional baseline for acute knee PJI in our center. It flags modifiable targets for preoperative optimization, supports a guideline-based prevention and surveillance algorithm and a prospective registry, and serves as a basis for future adequately powered multicenter studies.

## References

[1] Tande AJ, Patel R (2014). Prosthetic joint infection. Clin Microbiol Rev.

[2] Aftab MH, Joseph T, Almeida R, Sikhauli N, Pietrzak JR (2025). Periprosthetic joint infection: a multifaceted burden undermining arthroplasty success. Orthop Rev (Pavia).

[3] Osmon DR, Berbari EF, Berendt AR (2013). Diagnosis and management of prosthetic joint infection: clinical practice guidelines by the Infectious Diseases Society of America. Clin Infect Dis.

[4] Jämsen E, Nevalainen P, Eskelinen A, Huotari K, Kalliovalkama J, Moilanen T (2012). Obesity, diabetes, and preoperative hyperglycemia as predictors of periprosthetic joint infection: a single-center analysis of 7181 primary hip and knee replacements for osteoarthritis. J Bone Joint Surg Am.

[5] Parvizi J, Tan TL, Goswami K, Higuera C, Della Valle C, Chen AF, Shohat N (2018). The 2018 definition of periprosthetic hip and knee infection: an evidence-based and validated criteria. J Arthroplasty.

[6] Zimmerli W, Trampuz A, Ochsner PE (2004). Prosthetic-joint infections. N Engl J Med.

[7] Li C, Renz N, Trampuz A (2018). Management of periprosthetic joint infection. Hip Pelvis.

[8] Shohat N, Muhsen K, Gilat R, Rondon AJ, Chen AF, Parvizi J (2018). Inadequate glycemic control is associated with increased surgical site infection in total joint arthroplasty: a systematic review and meta-analysis. J Arthroplasty.

[9] Pulido L, Ghanem E, Joshi A, Purtill JJ, Parvizi J (2008). Periprosthetic joint infection: the incidence, timing, and predisposing factors. Clin Orthop Relat Res.

[10] Benito N, Mur I, Ribera A (2019). The different microbial etiology of prosthetic joint infections according to route of acquisition and time after prosthesis implantation, including the role of multidrug-resistant organisms. J Clin Med.

[11] Huotari K, Peltola M, Jämsen E (2015). The incidence of late prosthetic joint infections: a registry-based study of 112,708 primary hip and knee replacements. Acta Orthop.

[12] Franco-Cendejas R, Contreras-Córdova EL, Mondragón-Eguiluz JA, Vanegas-Rodríguez ES, Ilizaliturri-Sánchez VM, Galindo-Fraga A (2017). [Incidence of hip and knee prosthetic infections in a specialized center of Mexico City]. Cir Cir.

[13] Kong L, Cao J, Zhang Y, Ding W, Shen Y (2017). Risk factors for periprosthetic joint infection following primary total hip or knee arthroplasty: a meta-analysis. Int Wound J.

[14] Sandiford NA, Francescini M, Kendoff D (2021). The burden of prosthetic joint infection (PJI). Ann Joint.

[15] Shahi A, Parvizi J, Kazarian GS (2016). The alpha-defensin test for periprosthetic joint infections is not affected by prior antibiotic administration. Clin Orthop Relat Res.

[16] Allegranzi B, Bischoff P, de Jonge S (2016). New WHO recommendations on preoperative measures for surgical site infection prevention: an evidence-based global perspective. Lancet Infect Dis.

[17] Shohat N, Parvizi J (2017). Prevention of periprosthetic joint infection: examining the recent guidelines. J Arthroplasty.

